# Assessment of the Economic Impact of Belimumab for the Treatment of Systemic Lupus Erythematosus in the Italian Setting: A Cost-Effectiveness Analysis

**DOI:** 10.1371/journal.pone.0140843

**Published:** 2015-10-21

**Authors:** Francesca Pierotti, Ilaria Palla, Maarten Treur, Lara Pippo, Giuseppe Turchetti

**Affiliations:** 1 Institute of Management, Scuola Superiore Sant'Anna, Pisa, Italy; 2 Pharmerit International, Rotterdam, The Netherlands; 3 GlaxoSmithKline, Verona, Italy; Institut National de la Santé et de la Recherche Médicale (INSERM), FRANCE

## Abstract

**Objective:**

The purpose of this analysis is to evaluate the cost-effectiveness of belimumab, a new biological treatment specifically developed for the treatment of Systemic Lupus Erythematosus (SLE), in the Italian setting. SLE is a chronic non-organ specific autoimmune disease characterized by a disregulation of the immune system that involves many organs and systems.

**Methods:**

A cost-effectiveness micro-simulation model with a lifetime horizon originally developed for the UK was adapted to the Italian setting. The analysis compared Standard of Care (SoC) alone vs belimumab plus SoC from a National Healthcare Service (NHS) and societal perspective. Health-economic consequences of treatments and organ damage progression were calculated. When available, Italian data were used, otherwise UK costs were converted using Purchasing Power Parities (PPPs). Utility values were based on the EQ-5D™ assessments in the belimumab clinical trials (BLISS 52 and 76). Results were discounted with 3% for costs and effects. A maximum belimumab treatment duration of 6 years was assumed and wastage costs were considered.

**Results:**

Cost per life year gained (Incremental Cost-Effectiveness Ratio, ICER) and cost per Quality Adjusted Life Year (QALY) (Incremental Cost-Utility Ratio, ICUR) were €22,990 and €32,859, respectively. These values reduced to €20,119 and €28,754, respectively, when indirect costs were included.

**Conclusions:**

It may be concluded that in the Italian setting and according to the guidelines of the Italian Association of Health Economics (IAHE), belimumab was shown to be cost-effective, in terms of both ICER and ICUR, (€25–40,000/QALY).

## Introduction

Systemic Lupus Erythematosus (SLE) is a disease with an important economic impact on both healthcare system and society [1]. SLE is a chronic non-organ specific autoimmune inflammatory disease and is characterized by a dysregulation of the immune system that involves many organs and systems. It affects about 28,500 people in Italy, especially women of childbearing age (female-male ratio 9:1) who may have a compromised functional state and a decreased quality of life. Patients affected by SLE face a mortality risk 2–5 times higher than the general population [2–5]. SLE is characterized by three patterns of activity: relapsing-remitting (RR), chronic active (CA), and long quiescent (LQ) and despite the standard of care (SoC) 50% of patients have an active disease [6–8].

Costs include the treatment of active disease, especially disease flares, the management of comorbidities and organ damage, and loss of productivity due to impaired physical and mental health of patients [9–15]. Understanding the economic implications of the disease is important to inform decision making in the allocation of healthcare resources [1,16]. The recent retrospective study LUCIE (Systemic LUpus Erythematosus Cost of Care In Europe Study), carried in 5 European countries (France, Germany, Italy, Spain and UK) on 427 patients followed for two years, shows that the average cost for a high severity patient is higher than that for a mild-moderate severity patient (€4,748 [SD 4,972] *vs* €2,650 [SD 3,488]). Costs of pharmaceutical treatments are €2,518 in high severity patients and €1,251 in mild-moderate severity patients, accounting for 53% and 47% of total costs, respectively. In particular, severe flares are identified as the major predictor of cost with an increase of €1,002 *per* flare [17].

Sutcliffe et al. [18] report total mean annual costs *per* patient of £7,913, 67% of which is due to productivity loss (UK costs as at 2001). The systematic review of Meacock et al [19] reports mean annual direct costs per patient with SLE in the US, Canada, Europe, and China ranging from $2,214 to $16,875 and mean annual indirect cost estimates ranging from $2,239 to $35,540 (all values are referred to year 2010). These data highlight the importance of reducing disease activity including flares, and slowing down disease progression to organ damage; which a major determinants of increased costs paid by the National Healthcare Service (NHS) and by society [20,21].

To date, scientific literature that analyzes the economic impact of lupus in Italy is lacking. The only available source is the Italian arm of the LUCIE study related to 96 patients (49 severe, mean age 42.9 ± 11.7 years, 85.4% females) followed in 4 rheumatologic centers specialized in the treatment of SLE.

The average annual direct costs of SLE in Italy amount to €2,513; minimum and maximum values (respectively €239 and €15,536) show large variations of the direct costs related to the different profiles of severity and disease activity. The mean direct medical costs are 1.4 times higher in severe patients than in non-severe patients (€2,905 *vs* €2,104, p = 0.031).

In a 2-year follow-up, the average costs for patients with flare are 2.4 times higher than those of patients without (€6,420 *vs* €2,718, p <0.001). The number of severe flares is related to costs (p = 0.0487), with an incremental cost *per* flare of €594,13 [22,23].

The aim of this study is to evaluate the cost-effectiveness of belimumab, a new biological treatment specifically developed for the SLE treatment, in the Italian context and in patients with a high level of disease activity. The cost effectiveness analysis compared belimumab added to standard of care (SoC), typically glucocorticoids and various immunosuppressants, mostly unlicensed for SLE, with SoC alone. Belimumab was approved in the United States and Europe in 2011 with two phase III studies, BLISS-52 and BLISS-76 [24–26]. Belimumab is indicated as an add-on treatment in adults with a positive autoantibody test whose disease is still highly active despite standard treatment (e.g., anti-dsDNA positive and low complement) with the exception of patients with severe active lupus nephritis or severe active central nervous system lupus [27].

Costs associated to Belimumab in USA are estimated to be $40,000 in first year of treatment and $35,000 in the subsequent years [28].

## Methods

### The model

The objective of this study was to perform a cost-effectiveness analysis of belimumab added to SoC relative to SoC alone. The model takes into account both life years gained and QALYs (Quality Adjusted Life Years).

A micro-simulation model with a lifetime horizon and one year cycle is appropriate to compare the complex and heterogeneous disease course of SLE patients. The model extrapolates disease activity (as measured by patients’ SELENA-SLEDAI scores) and glucocorticoid use data from the BLISS studies over a lifetime horizon, based on patterns observed in SLE natural history data and regression models developed by Watson et al [29]. The model then uses long-term disease activity, glucocorticoid dose and other patient characteristics to estimate long-term outcomes (organ damage and mortality), based on further regression models developed by Watson et al [29]. The cost-effectiveness model was developed for GlaxoSmithKline by Pharmerit International. The model has been adapted to the Italian setting by the authors.

Base case analysis follows an NHS perspective including direct medical costs of SLE related to adult patients with active disease, autoantibody-positive, with a high degree of disease activity (positive anti-dsDNA and low complement levels) despite SoC using euro 2011 cost data.

Considering the strong impact of SLE on the daily personal and professional activity of a patient, usually a young woman of childbearing age, an additional analysis from the societal perspective accounting for costs due to productivity losses is performed [30–32].

### Model assumptions and sources of data

**Fig 1:**
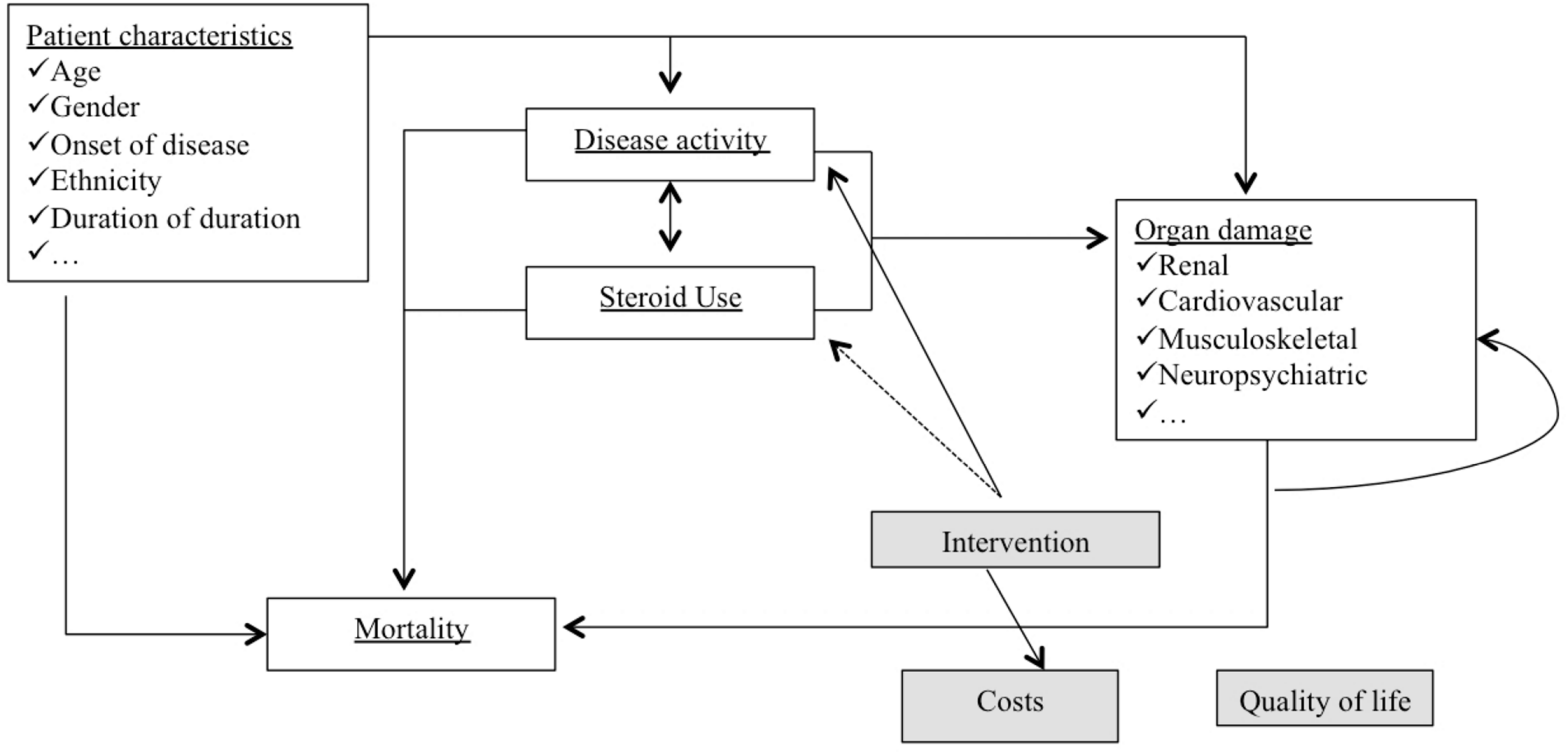
Modeled interdependencies between SLE related variables.

- Disease activity data during the first year was derived from the BLISS-52 and BLISS-76 trials results [24–26]. Thereafter a disease activity model was developed, using Johns Hopkins cohort natural history data, to estimate change in annual Adjusted Mean SLEDAI (AMS) score between two sequential periods including covariates for baseline characteristics, age, previous mean SLEDAI score, previous treatment, increased DNA, low complement, and hematological involvement [29]. The Johns Hopkins cohort (based in Baltimore, USA) is one of the largest and most informative datasets for SLE patients and, at the beginning of 2010, it included a population of 2,047 patients. The sample is of 1,354 patients: all observations preceding 1992 (when the SLEDAI, SLE Disease Activity Index was published) and those with a follow-up period <24 months (not helpful for the estimation of long-term outcomes) were excluded.
- A steroid dose model was used to estimate the mean corticosteroid dose for patients based on their disease activity status.
- Further regression models based on the Johns Hopkins cohort data were developed to predict long-term outcomes (specifically, 12 different organ domain models and a mortality model were developed, taking into account modeled long-term disease activity and corticosteroid dose as well as patient demographics and other characteristics) [29].
- The model also took into consideration short-term outcomes of SLE disease activity, the specific effects related to the improvement in quality of life (QoL) and healthcare costs. Costs analyzed in the model were related to management of disease activity in the short term, long-term organ damage and belimumab treatment (Fig 1).

- SoC included the following drugs, alone or in combination: corticosteroids (prednisone or equivalents, up to 40 mg/day), immunosuppressive or immunomodulatory agents (methotrexate, azathioprine), leflunomide, mycophenolate (mycophenolate mofetil, mycophenolate mofetil hydrochloride and sodium), calcineurin inhibitors (tacrolimus, cyclosporine), sirolimus, cyclophosphamide, 6-mercaptopurine, thalidomide, antimalarials (hydroxychloroquine, chloroquine, quinacrine), and NSAIDs (Non-Steroidal Anti-Inflammatory Drugs).
- Patients who do not respond to treatment with belimumab after 24 weeks (including those who do not follow responder criteria and those who discontinue treatment for other reasons, e.g. non-compliance, side effects, etc.) were switched to SoC. The response is measured in terms of reduction of the SELENA-SLEDAI (SS, Safety of Estrogens in Lupus Erythematosus National Assessment SLE Disease Activity Index) score ≥4 points relative to baseline. The choice of responder criteria is related to the primary endpoint in BLISS-52 and BLISS-76 trials, i.e. a response at week 52, defined as a reduction ≥4 points in SELENA-SLEDAI score, no worsening of Physicians Global Assessment (PGA <0.3 points), and no new-onset British Isles Lupus Assessment Group (BILAG) A or no more than one new-onset BILAG B.
- The rate of belimumab discontinuation was based on the BLISS trials and was due to loss of efficacy, lack of compliance or adverse events. Moreover, it was stratified by responder status.
- The belimumab additional effect to standard therapy after 52 weeks was assumed to remain constant throughout the whole treatment duration (six years).
- A discount rate of 3% was applied to costs and effectiveness according to the economic evaluation Italian guidelines [33].

**Fig 2:**
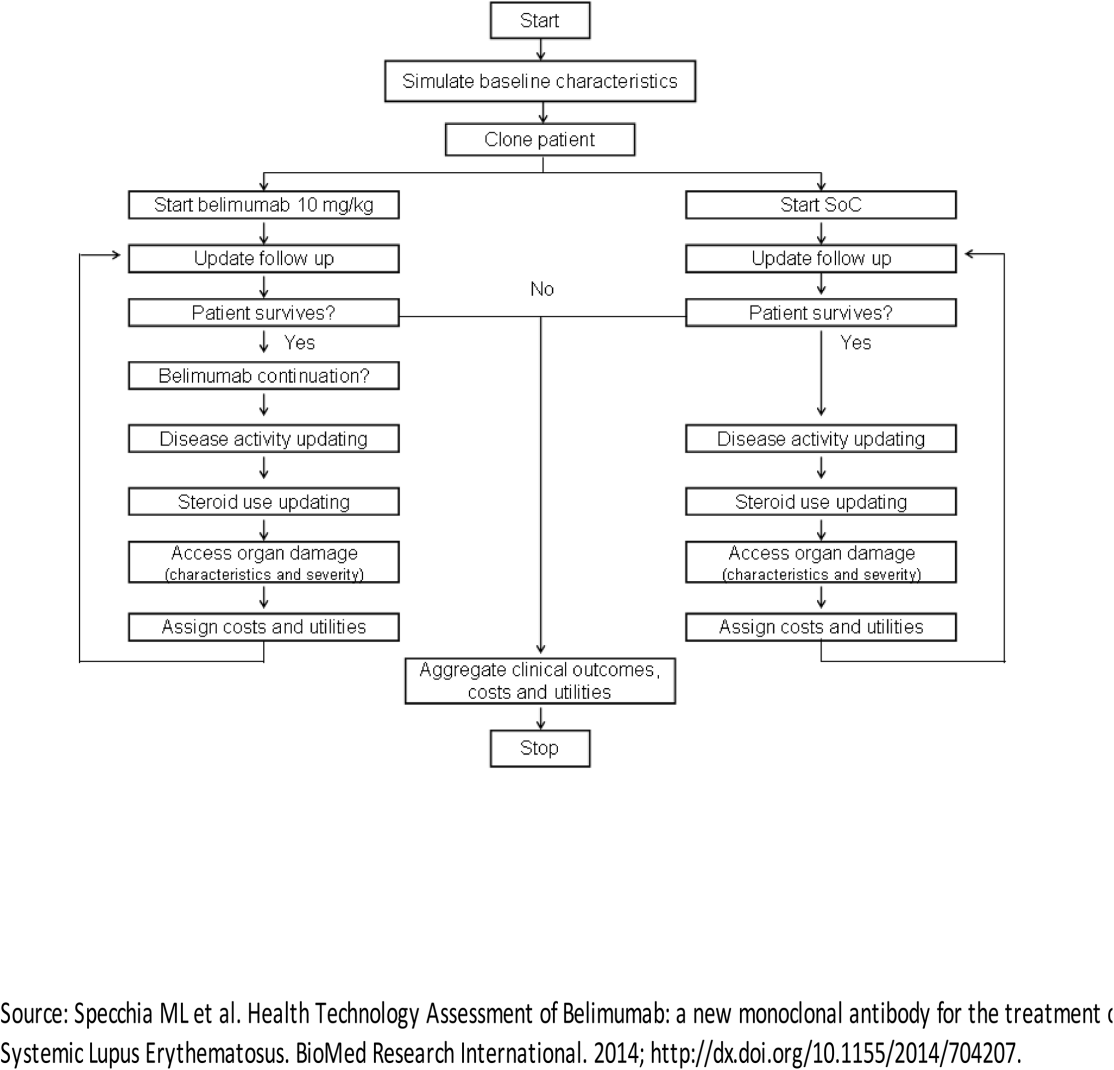
Schematic SLE patients flowchart used in the model.

The patients flow simulation is presented in Fig 2: for each year and for each treatment arm, the model analyzed whether the patient survives or dies. If the patient survives he/she could or could not continue the treatment with belimumab. The patient can stop the treatment for 3 reasons: natural discontinuation (loss of efficacy, no compliance); insufficient response to treatment, maximum duration of treatment reached. Disease activity (SELENA SLEDAI score), use of corticosteroids and accrual of organ damage are updated whether or not the patient continues the therapy with belimumab. Moreover, in the standard therapy arm, for the surviving patients, the activity of the disease, the use of corticosteroids and the possible organ damage were updated. Average costs, utilities and clinical outcomes were assigned to both arms for each cycle, and the process was iterated until the patient’s death.

### Data

#### Population

Baseline characteristics of the patients were obtained from the BLISS trials (Table 1). The simulation is based on 50,000 patients, the setting of the baseline simulation is founded on these assumptions: the maximum treatment duration with belimumab was set at six years based on the availability of six years of Belimumab phase II long-term open label safety and efficacy extension data at the time of the model development (seven years now published) [34], the duration of waning of belimumab effect was zero years and the scenario was with waste of product on the basis of drug prescription. The subgroup of patients with low complement and anti-dsDNA levels was included in the analysis, and the criterion of response is a reduction in SELENA SLEDAI score ≥4 at week 24. Mortality rates for general population by gender and age were extracted from mortality tables of the Italian population [35].

**Table 1:** Base case scenario: patients characteristics (based on BLISS trials).

Parameter	Value
Age at baseline (years)	34.7
Gender (% females)	93.9%
SLE^a^ disease duration (years)	6.6
Age at diagnosis (years)	28.1
Ethnicity (% afro-caribbean)	7.3%
SLICC^b^ score	0.61
SELENA-SLEDAI (SS)^c^ score	10.9
Daily steroids use (mg/day)	11.6

^*a*^ *SLE*: Systemic Lupus Erythematosus

^b^ *SLICC*: Systemic Lupus International Collaborating Clinics Damage Index

^c^ *SELENA-SLEDAI* (SS): Safety of Estrogen in Lupus Erythematosus National Assessment SLE Disease Activity Index

Source: Specchia ML et al. Health Technology Assessment of Belimumab: a new monoclonal antibody for the treatment of Systemic Lupus Erythematosus. BioMed Research International. 2014; http://dx.doi.org/10.1155/2014/704207

#### Costs

The model was adapted to the Italian setting using available costs and tariffs data related to Italy. Unit values of the Italian Healthcare Service (IHS) were extracted from the National Tariffs List of outpatient specialist care of the Italian Ministry of Health [36]. Most recent available tariffs have been used. All costs were converted into Euro 2010 price level using the Organisation for Economic Co-operation and Development’s (OECD) consumer price index, values expressed in pounds (from the original UK model) were converted based on the purchasing power of Euro-Sterling using Purchasing Power Parities (PPPs) for Healthcare (http://stats.oecd.org, values at 21 September 2011). In the model, cost data were related to the year of the event and to the following years.

#### Short term Disease activity Costs

The SELENA-SLEDAI score measures the disease activity at the time of the visit or in the previous 10 days; this time interval corresponds to the short-term disease activity. The relationship between costs and SELENA-SLEDAI score was obtained from values of the phase II study on belimumab LBSL02/99 [37]. The study reports the use of health care resources for the treatment of SLE as the number of surgical procedures, days of hospitalization, number of visits, diagnostic procedures performed by patients, etc. The estimated average short term cost for all SELENA-SLEDAI scores included in the model is €512.94 *per* patient/year. Among the short-term costs the costs related to the acquisition, administration and clinical monitoring of belimumab, were included. Since organ damage costs are considered separately, the short term cost model does not include a certain number of variables, e.g. the number of surgical interventions, visits to the emergency room, hospitalizations and home care assistance.

#### Belimumab Costs

The ex-factory price of belimumab amounts to €131.96 for the 120 mg vial and €439.88 for the 400 mg vial. According to NICE standards, the price used in the cost-effectiveness analysis is the ex-factory price with the application of a confidential patient access scheme (PAS) agreed between AIFA (Italian Medicines Agency) and the manufacturer. The annual cost of belimumab is calculated on the basis of patients’ weight distribution in the BLISS trials (for the specified subgroup). To obtain a realistic scenario, the analysis takes into account vial waste and it calculates vial cost per patient considering optimal vial combination.

#### Organ damage Costs

Organ damage costs were determined on the base of SLICC (Systemic Lupus International Collaborating Clinics) score data available in scientific literature. This was carried out on Pubmed database focusing on Italian articles. Since the available scientific evidence is poor, Italian costs were available only for some types of organ damage [38–43] and where data were absent, UK data were used. The cost for each type of organ damage was estimated as a weighted average; the weights system was based on the events of the Johns Hopkins cohort and it was calculated on the average number of events *per* year and *per* patient with a damage in a given organ system.

Some organ damage data were referred to the year of the event onset and to the following years. If data were not available for several consecutive years, costs are left constant. The Italian model, similarly to the UK model, assumed that skin damage and premature gonadal failure do not involve additional healthcare costs [44].

#### Indirect Costs

Indirect costs considered loss or decreased productivity due to the disease. The model estimated the annual cost of absenteeism, based on the hourly wage and the employment rate of the population by gender and age. The gross hourly wage by gender and age was extracted from the Statistical Observatory of companies (Italian Institute of Social Assistance) [42]. The study by Sutcliffe et al. [18] presented a model to estimate indirect costs based on disease activity score. By combining the data from this study with the average salary in the year of study and disease activity score (adjusted as the score used in the study is different by SLEDAI) it was possible to estimate the increase in hours missed from work for every unit of SELENA SLEDAI.

Indirect costs were estimated with the human capital approach including costs due to absenteeism and to death before retirement age (65 years). For patients died before reaching retirement age, the annual salary multiplied by the global SLE specific employment rate (46%) was applied, as reported by Briggs [45].

#### Quality of life

Utility values were calculated according to the scores of the EQ-5D™ questionnaire administered to the patients enrolled in the BLISS trials on days 28, 56, 84, 140, 168, 224, 252, 280, 336, 364 (BLISS-52 only) and on days 476, 532 (BLISS-76 only). EQ-5D results were converted into utility values using the algorithm of Dolan [44] that transforms values obtained from questionnaire into QoL values, using the time trade-off method from a representative sample of the UK population. The average utility for patients enrolled in the trials amounts to 0.70. Then, a linear regression was used to explain the relationships (if significant) between utilities and SELENA-SLEDAI score, organ damage and patient characteristics derived from the BLISS trials. This model allowed to estimate a net utility, free from organ damage effects. The values of disutility for organ damage, as well as for costs, were obtained from literature (NICE and Pubmed) for each of the 41 items of the SLICC score. The model predicted the yearly damage for each involved organ. The criterion for the system of weights is the same used for the costs. QoL values for patient’s organ damage were obtained by multiplying their net utility (based on SELENA SLEDAI score) by an organ damage disutility multiplier. If patients had several forms of organ damage, the lowest disutility multiplier was applied. The study used UK data in absence of Italian specific values setting.

### Scenario Analysis

Analyses of 4 alternative scenarios were performed:

1. scenario including indirect costs;
2. scenario without waste of product;
3. scenario with maxiumum belimumab treatment duration of 10 years;
4. scenarios with belimumab effects decreasing over time.

### Sensitivity Analysis

Probabilistic Sensitivity Analysis (PSA) was performed varying *per* 1,000 iterations the following parameters of the model, in parentheses the probability distribution assigned [45]:

- coefficients of week 52 change in SELENA SLEDAI score regressions (multivariate normal),
- coefficients of NHM for change in SELENA SLEDAI (multivariate normal),
- discontinuation rate (normal),
- response probability (gamma),
- coefficients of the NHM for mortality and organ damage development (multivariate normal),
- standardized mortality rates (normal),
- coefficients for BLISS utility regression (multivariate normal),
- costs associated with SELENA SLEDAI scores for first and subsequent years (gamma),
- organ damage costs (gamma),
- organ damage disutility (gamma),
- indirect costs (normal).

Univariate sensitivity analysis was performed for the same variables as outlined for PSA by setting each individual parameters at their 2.5% lower and 97.5% upper values to obtain a reference interval.

## Results

### Base case scenario

**Fig 3:**
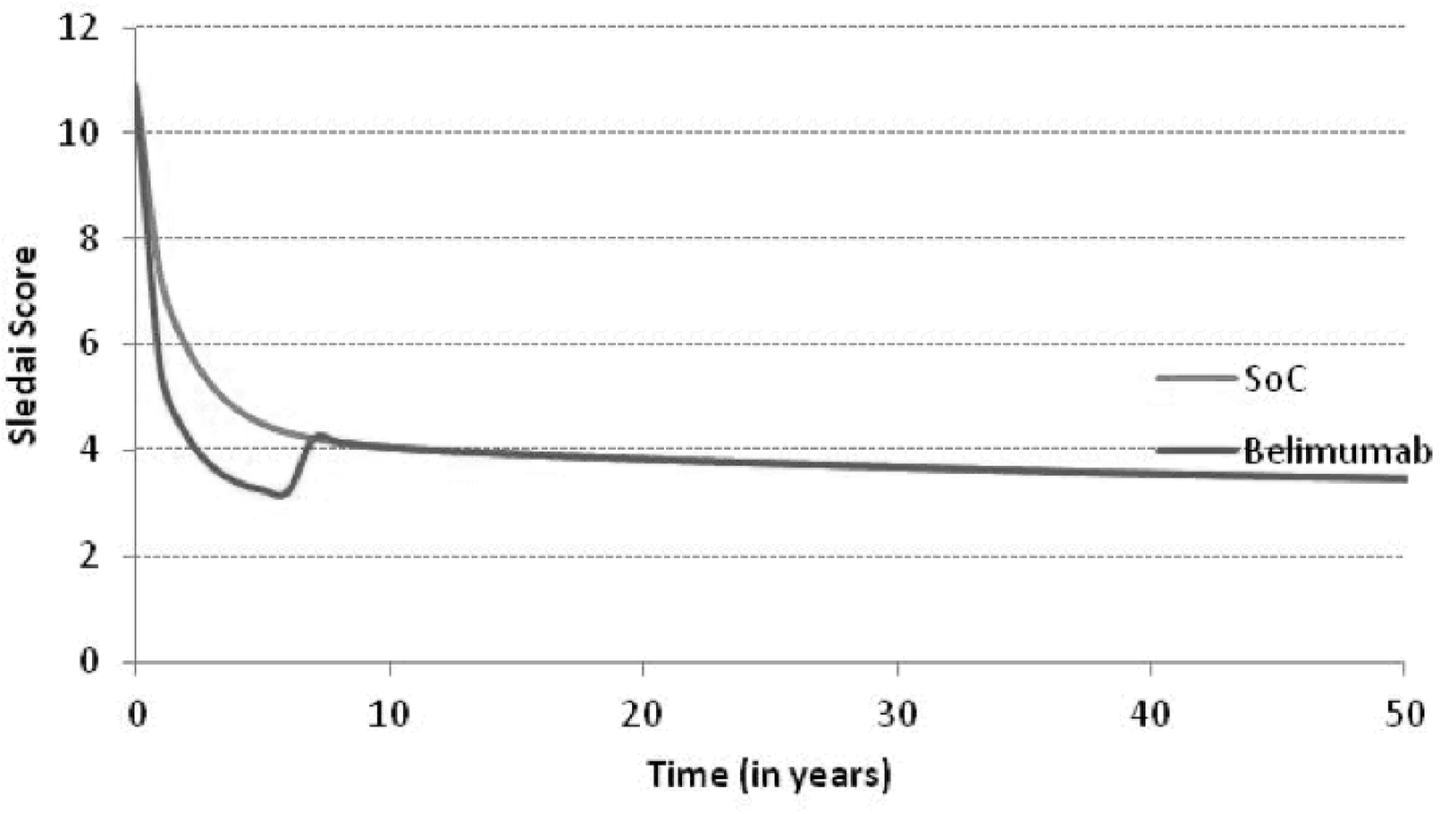
Average SLEDAI time course.

**Fig 4:**
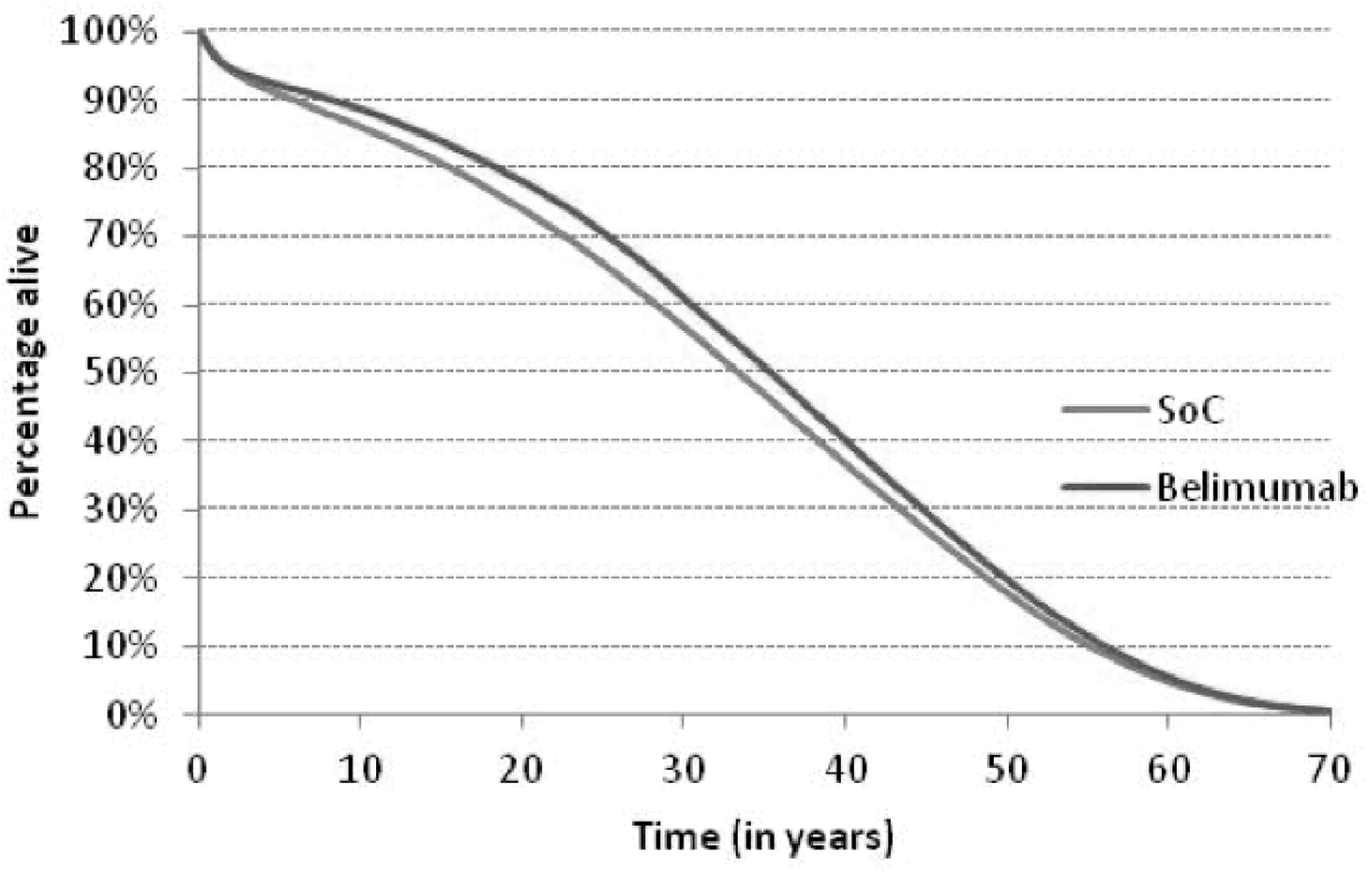
Survival time course.

Fig 3 shows the time course of the SELENA SLEDAI score with SoC compared to SoC *plus* belimumab. The analysis presents a greater reduction in SELENA-SLEDAI score in patients treated with belimumab compared to patients treated with standard therapy. The difference between the two arms decreases over time, due to the rate of belimumab discontinuation. After discontinuation patients get treated with SoC. This directly affected SLEDAI score and involvement parameters by applying the SoC effects. The reduction of the disease activity for patients treated with belimumab entails a reduction of corticosteroid doses and therefore of the expected risk of organ damage and mortality, with a potential consequent positive impact on survival (Fig 4). As patients treated with belimumab are projected to live longer than those treated with standard therapy, this implies a longer period of exposure to organ damage. In terms of organ damage, belimumab patients had on one hand a prolonged exposure to the risk, on the other hand a decreased disease activity (Table 2). Fewer patients on belimumab were predicted to develop cardiovascular, peripheral vascular, pulmonary and renal damage as well as premature gonadal failure compared to SoC. These results can be explained by the lower average disease activity for patients on belimumab compared to SoC and the dependence of damage risk on disease activity. On the contrary, more belimumab patients were predicted to develop damage than SoC for diabetes, gastrointestinal malignancy, musculoskeletal and ocular; this is justified by the increased life expectancy of patients treated with belimumab.

**Table 2:** Organ damage occurrence in SLE patients until death.

Organ damage	Standard therapy	Belimumab	Difference
Cardiovascular	30.1%	28.9%	-1.2%
Diabetes	19.0%	19.9%	0.9%
Gastrointestinal	22.2%	24.3%	2.0%
Malignancy	36.3%	38.0%	1.8%
Musculoskeletal	64.5%	65.2%	0.6%
Neuropsychiatric	46.9%	50.8%	0.9%
Ocular	60.5%	61.9%	1.4%
Peripheral vascular	20.9%	20.4%	-0.5%
Premature gonadal failure	6.9%	6.8%	-0.1%
Pulmonary	34.3%	33.3%	-1.0%
Renal	26.0%	22.4%	-3.6%
Skin	7.6%	7.5%	-0.1%

An important implication for the reduction of disease activity with belimumab is the delay in organ damage progression with a positive impact on patients’ QoL. Table 3 shows treatment and organ damage costs *per* patient in the two arms of intervention. The difference in terms of direct healthcare costs amount to €17,688. The additional cost of belimumab is partially counteracted by the lower treatment costs for organ damage. Table 4 reports the results in terms of cost *per* year of life gained (Incremental Cost-Effectiveness Ratio, ICER) and cost *per* QALY (Incremental Cost-Utility Ratio, ICUR): in the base case the ICER value is €22,990 and the ICUR value is €32,859. The base case analysis shows that belimumab is cost effective according to the thresholds suggested by IAHE guidelines (between € 25.000 e € 40.000 per QALY gained) and NICE parameters (between £20 000 and £30 000 for over 7 years) [46,47].

**Table 3:** Treatment and organ damage costs per patient.

Value (discounted)	Standard therapy	Belimumab	Difference
Short period	€ 8,228	€ 8,562	€ 333
Belimumab +administration	€ 0	€ 20,632	€ 20,632
Cardiovascular	€ 2,828	€ 2,722	€ 105
Diabetes	€ 5,934	€ 6,258	€ 324
Gastrointestinal	€ 523	€ 563	€ 40
Malignancy	€ 1,659	€ 1,706	€ 47
Musculoskeletal	€ 24,158	€ 24,742	€ 584
Neuropsychiatric	€ 9,325	€ 9,640	€ 315
Ocular	€ 576	€ 575	€ 0
Peripheral vascular	€ 1,928	€ 1,901	€ -27
Premature gonadal failure	€ 0	€ 0	€ 0
Pulmonary	€ 53,658	€ 51,636	€ -2,023
Renal	€ 16,418	€ 13,984	€ -2,433
Skin	€ 0	€ 0	€ 0
**Total direct costs**	**€ 125,234**	**€ 142,921**	**€ 17,688**
Costs due to absenteeism	€ 8,505	€ 7,452	€ -1,052
Costs caused by death	€ 7,281	€ 6,124	€ -1,157
**Total indirect costs**	**€ 15,786**	**€ 13,576**	**€ -2,210**
**Total costs**	**€ 141,019**	**€ 156,497**	**€ 15,478**

**Table 4:** Cost-effectiveness: base case results.

Value (discounted)	Standard therapy	Belimumab	Difference
Life years	18.99	19.76	0.77
QALYs^a^	10.78	11.31	0.538
Costs	€ 125,234	€ 142,921	€ 17,688
ICUR^b^			€ 32,859
ICER^c^			€ 22,990

^*a*^ *QALYs*: Quality Adjusted Life Years

^b^ *ICUR*: Incremental Cost-Utility Ratio

^c^ *ICER*: Incremental Cost-Effectiveness Ratio

### Alternative scenarios

Table 5 reports results obtained from alternative scenarios. If the productivity loss is considered, results change from baseline: the ICER decreases because, at the same effectiveness level, the difference in costs between the treatment arms decreases due to indirect costs. The largest impact of belimumab is on the costs due to death before the retirement age, as belimumab patients have a better life expectancy, less patients died before their retirement age. The effect of belimumab on absenteeism costs is relatively small as these costs are calculated based on the disease activity and also as no effects are included after six years. In the scenario without vial waste, ICER decreases in comparison to baseline as, at the same effectiveness level, the difference in costs decreases because the reduction of drug cost per administration of belimumab. With belimumab treatment duration of 10 years instead of 6 years, a slight increase in terms of effectiveness but also an increase in terms of therapy costs with belimumab is to be expected. This means an increase in the difference between costs and an higher ICER in comparison to the base case.

The base case analysis assumes a constant effect of belimumab during the 6 years of treatment, the last two scenarios showed the effect of belimumab remains maximal for 4 years, then it declines and remains similar to standard therapy, while in the other the effect is maximal for 5 years, then in the last year it declines towards the levels of the standard therapy. The results showed that the decline of belimumab towards the level of standard therapy in the last two or one year is not substantial.

**Table 5:** Cost-effectiveness: alternative scenario results.

**Scenario with indirect costs**			
Value (discounted)	Standard therapy	Belimumab	Difference
Life years	18.99	19.76	0.77
QALYs	10.78	11.31	0.538
Costs	€ 141,019	€ 156,497	€15,478
ICUR			€ 28,754
ICER			€ 20,119
**Scenario without waste of product**			
Value (discounted)	Standard therapy	Belimumab	Difference
Life years	18.99	19.76	0.77
QALYs	10.78	11.31	0.538
Costs	€ 125,234	€ 142,185	€ 16,951
ICUR			€ 31,491
ICER			€ 22,033
**Scenario with a belimumab treatment duration of 10 years**			
Value (discounted)	Standard therapy	Belimumab	Difference
Life years	18.99	19.87	0.87
QALYs	10.78	11.39	0.615
Costs	€ 125,234	€ 149,530	€ 24,296
ICUR			€ 39,515
ICER			€ 27,800
**Scenario with a decreasing belimumab effect over time (maximal 4 years + decreasing 2 years)**			
Value (discounted)	Standard therapy	Belimumab	Difference
Life years	18.99	19.68	0.69
QALYs	10.78	11.26	0.483
Costs	€ 125,234	€ 142,807	€ 17,573
ICUR			€ 36,372
ICER			€ 25,444
**Scenario with a decreasing belimumab effect over time (maximal 5 years + decreasing 1 year)**			
Value (discounted)	Standard therapy	Belimumab	Difference
Life years	18.99	19.71	0.72
QALYs	10.78	11.28	0.505
Costs	€ 125,234	€ 142,840	€ 17,607
ICUR			€ 34,878
ICER			€ 24,374

### Sensitivity analysis

**Fig 5:**
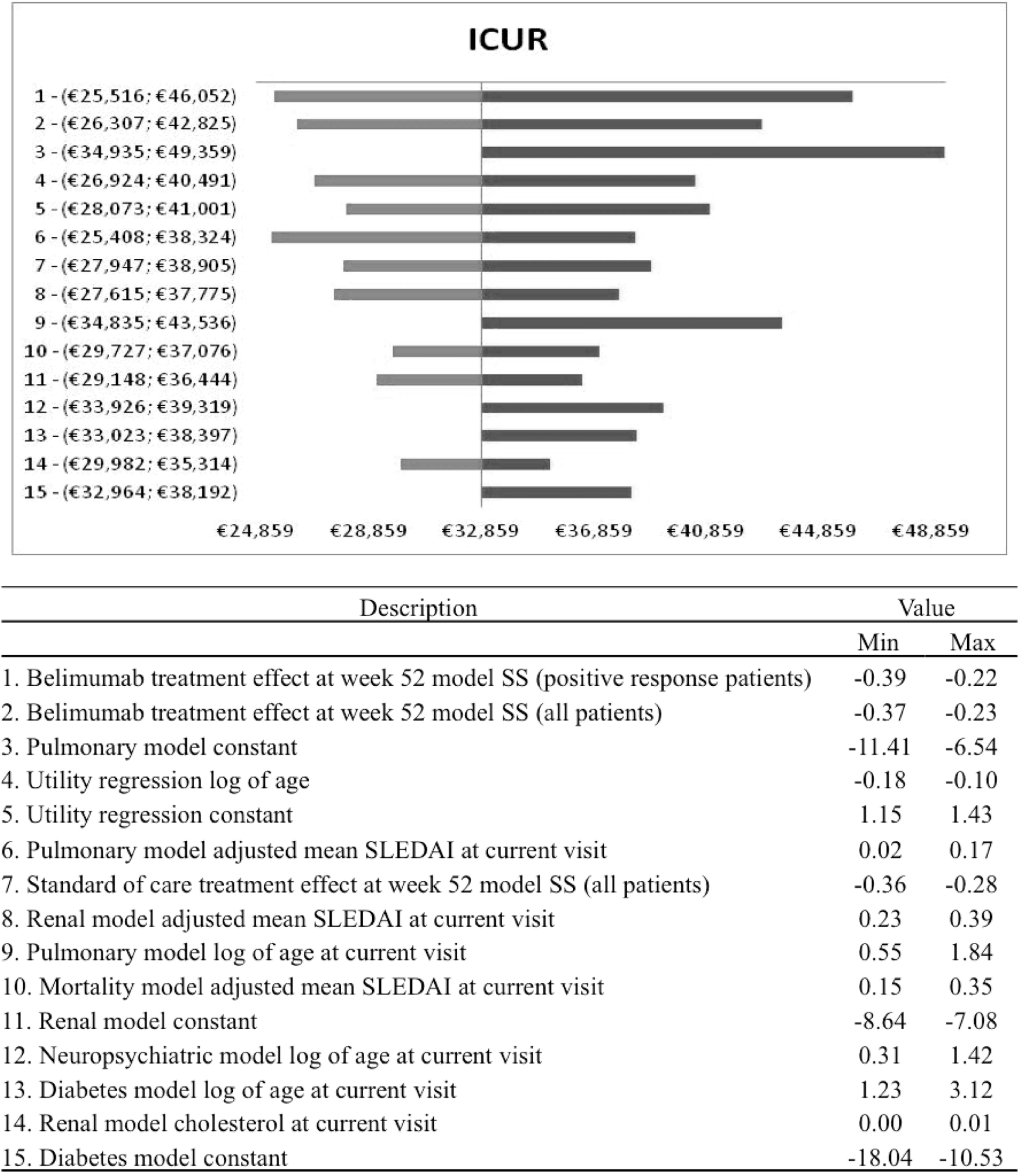
Univariate results: tornado diagram.

Taking into account the univariate sensitivity analysis, the effect of the treatment and the discontinuation rate are the main drivers of the model to determine the effect after 52 weeks. Fig 5 shows the *tornado* diagram for the ICUR with the corresponding 95% confidence intervals. The univariate analysis is conditional on keeping the other parameters fixed, this is not very likely due to the correlation between several model parameters. For this reason the interpretation of the univariate results should be cautious and combined with the results of PSA.

**Fig 6:**
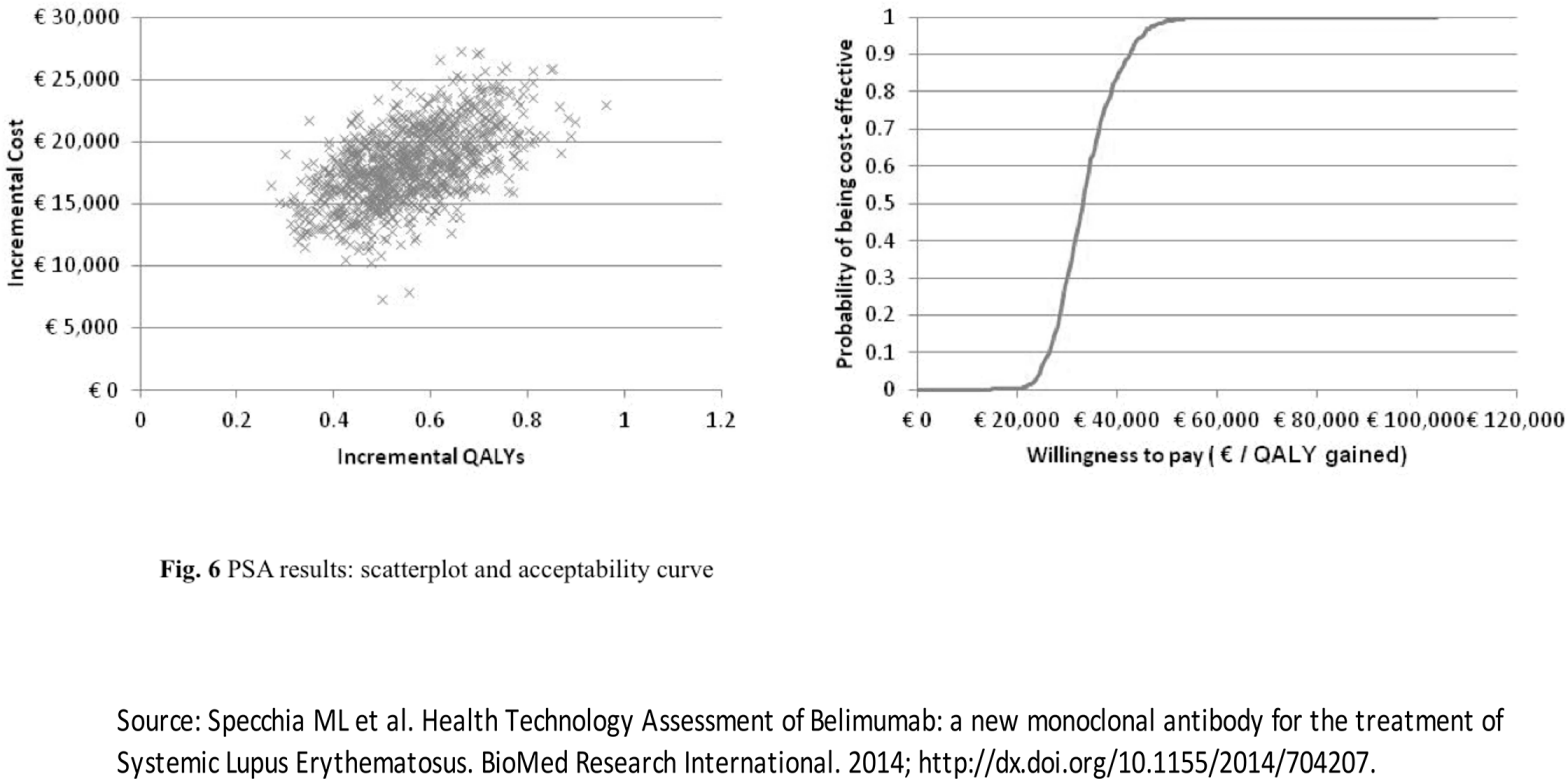
PSA results: scatterplot and acceptability course.

Fig 6 shows the scatter plot and the acceptability curve based on the probabilistic sensitivity analysis.

Results show that for a willingness to pay (WTP) of €30,000 *per* QALY gained, there is a probability of 29.1% that belimumab is cost-effective compared to the standard therapy for the selected patients’ group. The probability increases to 84.3%, when the WTP reaches the level of €40,000 *per* QALY gained.

## Conclusions

Systemic Lupus Erythematosus is a chronic non-organ specific autoimmune disease that may affect many organs and systems. Sustained disease activity and chronic therapy with corticosteroids and immunosuppressives can lead to organ damage and increased mortality. Belimumab is the first novel drug approved for SLE in over 50 years. The efficacy of belimumab added to SoC compared to SoC alone has been demonstrated in two large clinical trials.

This work studied the cost-effectiveness of belimumab in SLE using a microsimulation model originally developed for the UK setting. Recently, Díaz-Cerezo et al. [48] adapted the model to the Spanish context to assess the cost-effectiveness of belimumab plus standard of care versus the standard of care alone. The results show that the ICER was €16,647 per life year gained and the ICUR was €23,158 per additional QALY gained.

We have shown that in the Italian setting, and in an appropriate high disease activity sub-population of patients, Belimumab can be a cost-effective use of NHS resources similarly to evidence for Spain [48]. Furthermore, given the significant societal costs involved in this disease affecting mainly young women, we have shown that Belimumab may have positive economic consequences beyond the NHS perspective.

In this work belimumab was not compared to rituximab because the latter has not been registered for the treatment of SLE, since clinical studies [49] have not provided strong enough evidence. Currently, rituximab is used off-label in SLE and mainly in patients with active lupus nephritis, which is not the target population for belimumab.

The model has been designed based on clinical data, published literature and a number of other assumptions as is normally the case for recently approved medicines.

The analysis was based on referenced data and models, and, in some specific aspects, can be affected by the lack of country specific evidence. Therefore, a major effort of the working group was to validate and share each assumption of the model, which is the first developed for SLE and therefore is not comparable in terms of results with any previous study.

The base case analysis shows an ICER value *per* life year gained of €22,990 and an incremental cost *per* QALY of €32,859 based on a belimumab price which includes a confidential PAS agreed between AIFA and the drug’s manufacturer. Increased life expectancy and QALY were explained by lower Average Mean SLEDAI, decreased mortality risk and higher quality of life for patients on belimumab during 6 years of treatment. Lower disease activity was also associated with decreased risk of organ damage, as shown by fewer occurrences of cardiovascular, peripheral vascular, pulmonary, renal and premature gonadal failure. On the contrary, higher occurrences of diabetes, gastrointestinal malignancy, musculoskeletal and ocular were justified by the increased life expectancy and therefore prolonged exposure risk of patients treated with belimumab.

The cost-effectiveness model provided results that fall within an acceptable threshold, considering the low prevalence of the disease. These values are within acceptable threshold regions, implicitly or explicitly used in healthcare systems comparable to the Italian system, ranging between €25,000 and €40,000 *per* QALY [46]. According to these thresholds, belimumab is cost-effective compared to SoC. Given the complexity of the model and the underlying assumptions, results of other scenarios are presented to take into account, for example, a different treatment duration. Univariate sensitivity analysis showed that the main drivers of incremental results are the additional effect of the treatment with belimumab on disease activity and the discontinuation rate. If patients stay on belimumab for a longer time, more health gains and costs are incurred as showed also by scenario analysis results. Another scenario tested the assumption that additional effect on disease activity for belimumab compared to SoC remains constant over 6 years of treatment: results showed a slight decline of the effect of belimumab towards the level of standard therapy without affecting significantly cost effectiveness results.

PSA results show that for a willingness to pay of €30,000 *per* QALY gained, there is a probability of 29.1% that belimumab is cost-effective compared to the standard therapy for the selected patients’ group. The probability increases to 84.3%, when the WTP reaches the level of €40,000 *per* QALY gained.

As observed for other drugs used in rheumatologic diseases, further clinical and economic studies will be needed to accurately define all parameters and values used in the model and thus obtain more precise estimates of the cost-effectiveness [50–53].
